# Target Inhibition of CBP Induced Cell Senescence in BCR-ABL- T315I Mutant Chronic Myeloid Leukemia

**DOI:** 10.3389/fonc.2020.588641

**Published:** 2021-01-08

**Authors:** Ke Yang, Fang Wang, Hong Zhang, Xiaokun Wang, Likun Chen, Xiaodong Su, Xingping Wu, Qianqian Han, Zhen Chen, Zhe-Sheng Chen, Liwu Fu

**Affiliations:** ^1^ Sun Yat-sen University Cancer Center, State Key Laboratory of Oncology in Southern China, Collaborative Innovation Center for Cancer Medicine, Guangzhou, China; ^2^ Department of Research and Development, Guangzhou Handy Biotechnological Co., Ltd., Guangzhou, China; ^3^ Department of Pharmaceutical Sciences, College of Pharmacy and Health Sciences, St. John’s University, Queens, NY, United States

**Keywords:** CREB binding protein, cell differentiation, cell senescence, chronic myeloid leukemia, tyrosine kinase inhibitors resistance

## Abstract

The treatment of chronic myeloid leukemia (CML) with BCR-ABL tyrosine kinase inhibitors (TKIs), such as imatinib, has yielded clinical success. However, the direct targeting of BCR-ABL does not eradicate CML cells expressing mutant BCR-ABL, especially the T315I mutation in BCR-ABL. Moreover, increasing mutations were identified in BCR-ABL domain, resulting in TKIs resistance recently. It is necessary to find BCR-ABL-independent target for treating CML patients with various mutations, including T315I mutation in BCR-ABL. The dichotomous behavior of CREB binding protein (CBP) and E1A protein (p300), recruited by β-catenin associated with self-renewal and differentiation, have been identified in hematopoietic stem cells, respectively. In this study, CBP was aberrantly expressed in CML cells on the basis of Oncomine dataset. The β-catenin bound with much more CBP than p300 in CML cells. Down-regulation of CBP inhibited cell proliferation capacity and increased the binding of β-catenin to p300, thus promoting cell differentiation and p53-dependent cell senescence in CML cells with either wild type or T315I mutant BCR-ABL *in vitro* and *in vivo* models. These demonstrate CBP blockage can be developed for the treatment of CML independent of BCR-ABL mutation status including T315I.

## Introduction

Chronic myeloid leukemia (CML) is a clonal malignant disorder of pluripotent hematopoietic stem cells (HSCs), characterized by the presence of Philadelphia chromosome (Ph) translocation t(9;22)(q34;q11), leading to the formation of the novel BCR-ABL fusion gene (1). Despite BCR-ABL tyrosine kinase inhibitors (TKIs), such as imatinib, nilotinib, and dasatinib, have been developed, a significant number of patients develop resistance to treatment overtime (2, 3). The most frequent mechanism producing resistance is the increasing kinase domain mutations, notably the T315I mutation, which reduces or completely ablates drug efficacy (4–7). Recently, increasing new mutations have been discovered in BCR-ABL kinase domain, leading to TKIs resistance. Therefore, it is of great importance to find BCR-ABL-independent target for treating TKIs resistant CML patients, especially T315I mutation in BCR-ABL.

Despite the high degree of homology and similar patterns of expression, CBP and p300 play unique and distinct roles in gene regulation (8, 9). Distinct roles for CBP and p300 in hematopoietic stem cells (HSC) fate decisions have been identified (10, 11). The researchers described that CBP, but not p300, is crucial for HSC self-renewal, conversely, p300, but not CBP, is essential for hematopoietic differentiation. Multipotency and self-renewal capacities (12, 13) continuously provide differentiated cells, while properly maintaining the HSC pool size throughout life by precisely balancing differentiation and self-renewal (10). The activation of Wnt/β-catenin pathway recruits CREB banding protein (CBP) or E1A protein (p300) triggering cell self-renewal and differentiation in HSC, respectively (8, 10, 14). Aberrations in Wnt/β-catenin have been reported to have key roles in CML (15–17). Accumulating evidences reported that the Wnt pathway is activated and promotes cell proliferation and survival, conferring resistance to the TKIs (15, 17, 18). Therefore, we deduced that the effective inhibition of CBP might disrupt the ectopic balance between β-catenin/CBP and β-catenin/p300 complexes and result in cell differentiation and senescence in CML.

In the current study, more p300 is bound to beta-catenin when CBP is knock-down. Down-regulation of CBP increased the binding of β-catenin to p300, thus promoting cell differentiation and p53-dependent cell senescence and inhibiting cell proliferation capacity in CML cells with either wild type or T315I mutant BCR-ABL *in vitro* and *in vivo* models.

## Materials and Methods

### Chemicals and Antibodies

Nitrobluetetrazolium (NBT) and phorbol 12-myristate 13-acetate (PMA) were purchased from Sigma-Aldrich (St. Louis, MO, USA). Antibodies against β-catenin (#8480), p21(#2947S), phosphorylated Rb (#9307S), and CyclinD1 (#2922S) were purchased from Cell Signaling Technology (Danvers, MA, USA). CBP (sc-369), p300 (sc-584), JUN (sc-45) were purchased from Santa Cruz Biotechnology Inc. (Paso Robles, CA, USA). p16 (ab117443) and recombinant mouse Wnt3a protein was purchased from Abcam (Cambridge, MA,USA). Glyceraldehyde-3-phosphate dehydrogenase (GAPDH) antibody (822051) was purchased from Kangchen Co. (Shanghai, China). Cell protein Extraction Kit and Nucleoprotein Extraction Kit was purchased from Beyotime (Shanghai, China). The senescence detection kit was purchased from Beyotime (Guangzhou, China).

### Cell Culture

The human CML cell line K562 was obtained from ATCC and the murine 32D myeloid cells stably expressing either wild-type BCR-ABL (32D-WT) or T315I mutant BCR-ABL (32D-T315I) were obtained from Professor Pan JX (2014, Ophthalmic Hospital, Sun Yat-sen University, Guangzhou China). BCR-ABL expression in these cells was checked by Western-blot for verification every 3 to 4 months routinely. Cells were maintained in RPMI1640 medium supplemented with 10% fetal bovine serum (FBS, Gibco), 1 unit/ml penicillin and 1 mg/ml streptomycin at 37°C and 5% CO2, 32D-WT, and 32D-T315I cells were additionally supplemented with 0.5 ng/ml WNT3a to imitate the feature of activated Wnt signaling in CML cells.

### RNA Interference Transfection

For depletion of CBP and p300, the pSIH-H1-puro-construct (System Bioscience, SBI) containing CBP or p300 short hairpin RNAs (shRNA) was generated by cloning the following CBP specific or p300 specific RNAi target sequences into pSIHF-H1:

CBP shRNA: 5-GATCCCGTTTACCATGAGATCCTTATCTTCCTGTCAGAATAAGGATCTCATGGTAAACGTTTTTG-3’.

P300 shRNA:5’- GATCCTAACCAATGGTGGTGATATTACTTCCTGTCAGATAATATCACCACCATTGGTTATTTTTG-3’.

Briefly, CMLs silencing CBP or p300 were generated by infection with CBP shRNA lentiviruses or p300 shRNA lentiviruses, and selected by treatment with 0.5 μg/ml puromycin for 10 days, beginning from 48 h after infection.

### Nitro Blue Tetrazolium Assay

K562, 32D-WT, and 32D-T315I cells receiving different treatment were cultured in 96-well plates for 72 h. The NBT reduction assay was performed as Collins’description (19).

### SA-ß-Galactosidase Assay

For the SA-ß–galactosidase assay, the SA-ß-galactosidase activity was determined using a senescence detection kit (Beyotime) according to the manufacturer’s instructions. Senescent cells were identified as blue-stained cells under an Olympus IX71S8F inverted microscope.

### Western Blot Analysis

Cell specimens were washed twice with PBS and total cellular protein was extracted with RIPA. Nuclear proteins were extracted by nucleoprotein extraction buffer and were used when detecting expression of ß-catenin. Equal amounts of protein (50–100 ug) were separated on sodium dodecyl sulfate-polyacrylamide gel electrophoresis, and then transferred to PVDF membranes, blocked and incubated overnight with primary antibodies at 4°C. After the secondary monoclonal antibodywas applied to visualize proteins using Kodak X-AR film, the results were analyzed by Gel-Pro Analyzer.

### Mice Xenograft Models

All mouse experiments were performed according to protocols approved by the Institutional Animal Care and Use Committee of Sun Yat-Sen University. Female BALB/c nude mice, age of 8-week-old, weighing 18-22g were purchased from the Center of Experimental Animal of Guangzhou University of Chinese Medicine (Guangzhou, China). To test the incidence of tumors and proliferation ability, 32D-WT or 32D-T315I cells with CBP or p300 knockdown were subcutaneously injected into left subaxillary of mice, respectively. Tumor volume (mm^3^) was calculated as L×W^2^×0.5 (L indicates the length-diameter and W the width-diameter of the tumor) (10).

### Co-Immunoprecipitation Assay

Total nucleoprotein lysates were extrcated and prepared by ice-cold IP lysis buffer (Beyotime, China), and the supernatant of cell lysate corresponding to1mg of total protein was precleared by Protein G Plus/Protein A-agarose beads (Millipore, USA) to minimize nonspecific binding, followed by the addition of either mouse IgG (Beyotime, China) or β-catenin overnight. The bound proteins were washed thrice with lysis buffer and dissociated with the beads *via* boiling and centrifugation. The immnoprecipitated proteins were then analyzed by standard immune blotting (10).

### Statistics

Data analysis was performed using the SPSS 16.0 software package. All experiments were repeated at least three times, and data are presented as Means ± SEM. The mean values were compared using unpaired student’s t-test (two groups). A value of *p*<0.05 was considered statistically significant.

The authenticity of this article has been validated by uploading the key raw data onto the Research Data Deposit public platform (www.researchdata.org.cn), with the approval RDD number as RDDB2020000942.

## Results

### Silencing Endogenous CBP Promotes Cell Differentiation and Senescence in CMLs Independent of the Mutation Status of BCR-ABL

**Figure 1:**
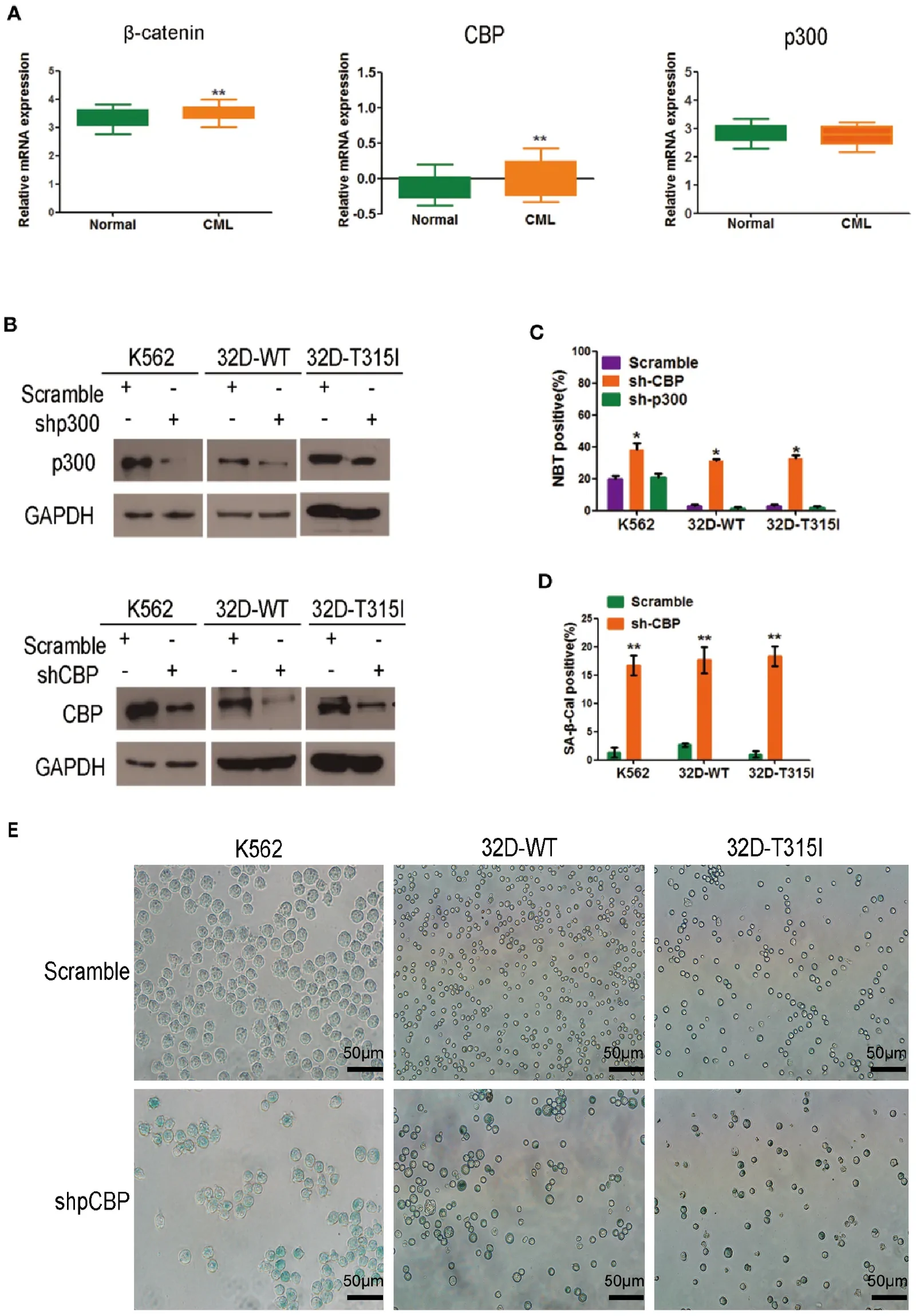
Effect of CREB binding protein (CBP) down-regulation on cell differentiation and scenescence in chronic myeloid leukemia (CMLs) expressing wild-type or T315I mutant BCR-ABL. **(A)** *In silico* analysis of β-catenin, CBP, and p300 expression in human CML. Data was analyzed using publicly available Oncomine database (www.oncomine.org); *p < 0.05; **p < 0.01 (Student’s t-test). **(B)** Efficient knocks down CBP or p300 in K562, 32D-WT, 32D-T315I cells as revealed by Western blot. **(C)** Differentiation status was determined by nitrobluetetrazolium (NBT) reduction assay in K562, 32D-WT, and 32D-T315I cells following the down-regulation of CBP or p300. **(D)** Cell senescence status was determined in cells with the indicated vector (scramble and sh-CBP) transfection for 72 h by the SA-β-Gal staining assay. The values are evaluated using the SA-β-Gal positive ratio. **(E)** Senescent cells were identified as blue-stained cells under an Olympus IX71 inverted microscope. Representative images of senescent cells were taken by Olympus IX71 inverted microscope. The data represent Mean ± SEM derived from three independent experiments. Signiﬁcant changes are indicated as follows: *p < 0.05; **p < 0.01, compared to scramble group (unpaired student’s t-test).

To understand the key roles of CBP and p300 in CMLs, we analyzed these genes using the available Oncomine datasets. The data from the Haferlach Leukemia Statistics (Necleotid acc. No. U85962) showed that CBP (*p*=0.002) and β-catenin (*p*=0.001) expression was significantly up-regulated in 76 cases of CML samples compared with 74 cases of normal samples (**Figure 1A**). However, p300 expression was not apparently altered compared to the normal samples (*p*=0.900), suggesting that β-catenin/CBP is preferentially expressed in malignant proliferative CMLs. To further evaluate the significance of CBP and p300 on cell growth or differentiation in CMLs, we silenced endogenous CBP and p300 using lentiviral short -hairpin RNA (shRNA) in K562, 32D-WT, and 32D-T315Icells, respectively (**Figure 1B**) and detected the status of cell differentiation by the NBT reduction assay. As is shown in **Figure 1C**, in K562, 32D-WT, and 32D-T315 CML cells, the ratio of differentiated cells after CBP silence was higher than those scramble cells. In contrast, silence of p300 did not change the differentiate ratio compared with the scramble cells, implying that silencing endogenous CBP rather than p300 have the significant potential to promote cell differentiation in CMLs independent of BCR-ABL mutation status.

Furthermore, the SA-β Gal staining was performed to verify the degree of cell differentiation. Notably, a significant enlarged and flattened morphology was observed in CBP silenced CMLs, indicating a senescent appearance (**Figure 1E**). The results demonstrated that the CBP silenced CMLs showed a higher ratio of senescent cell in comparison with those control cells, suggesting that CBP knockdown-induced differentiated cells acquired senescence property in BCR-ABL wild-type and mutant CMLs (**Figure 1D**).

### Knockdown of CBP Inhibited Cell Proliferation and Tumor Growth *In Vitro* and *In Vivo*

**Figure 2:**
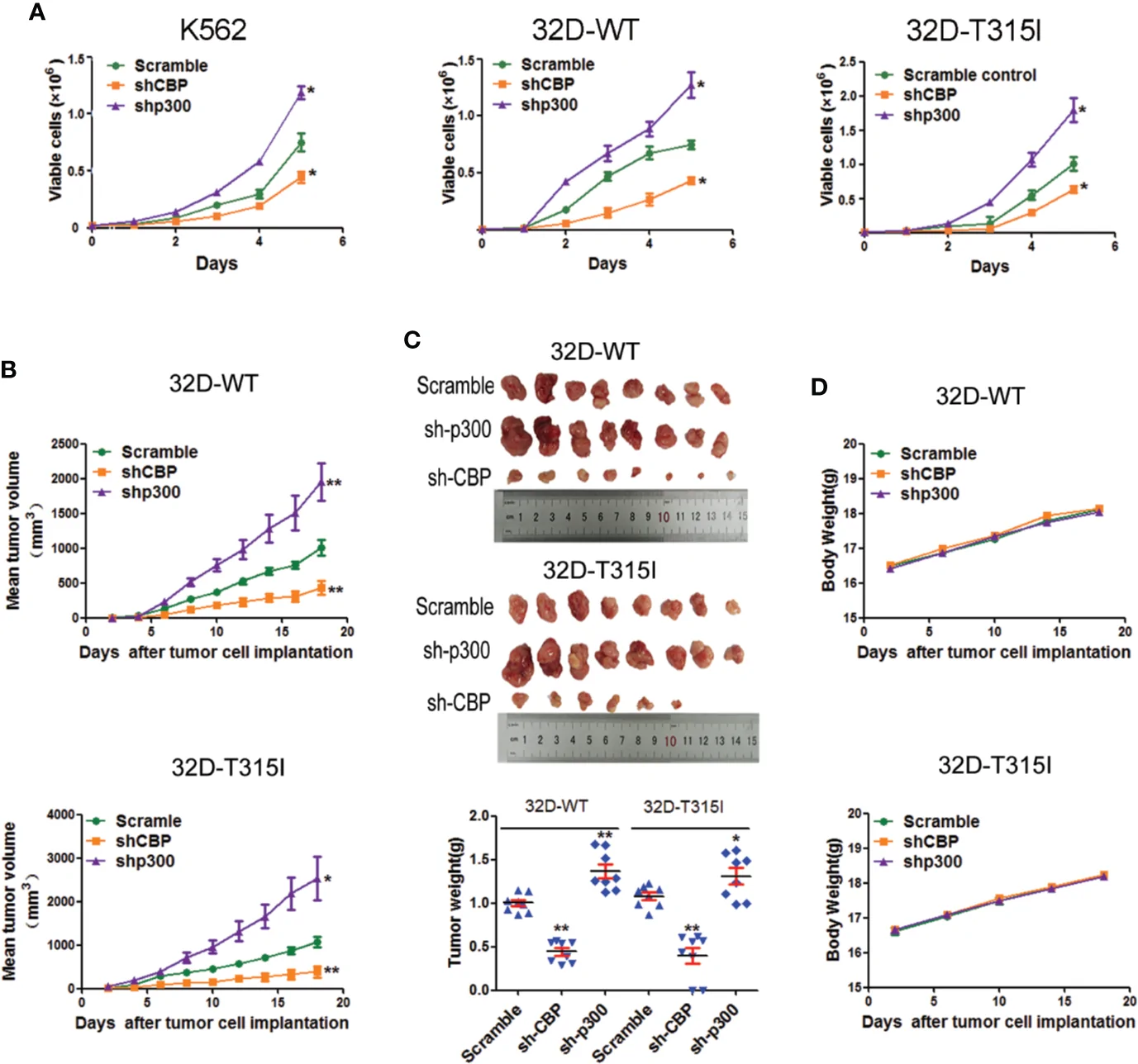
Knock down of CREB binding protein (CBP) inhibited cell proliferation *in vitro* and *in vivo*. **(A)** The proliferation ability was assessed in chronic myeloid leukemia (CMLs) by cell counting and viable cell number was determined by using the trypan blue exclusion assay. The data represent Mean ± SEM derived from three independent experiments. **(B)** The nude mice xenotransplantation assay. The tumor growth curve was drawn to monitor the tumor volume over time. Tumor volume (mm3) was calculated as L×W2×0.5 (L indicates the length-diameter and W the width-diameter of the tumor). **(C)** Representative images of tumors from nude mice that received subcutaneous injections of the indicated cells were shown. Tumor weight was analyzed after implantation. **(D)** The change of body weight was measured in nude mice. Signiﬁcant changes are indicated as follows: *p < 0.05; **p < 0.01, compared to scramble group (unpaired student’s t-test).

To further validate the significance of CBP and p300 on cell growth, *in vitro* and *in vivo* assays were performed in CMLs. As anticipated, the silence of CBP induced a significant inhibition of cell proliferation compared with the vector control, independent of the mutation status of BCR-ABL such as T315I, whereas knockdown of p300 primarily accelerated cell proliferation (**Figure 2A**). Consistent with the *in vitro* results, CBP knockdown significantly impaired tumor growth and reduced the tumor weight (**Figures 2B, C**), in contrast, silence of p300 increased tumor formation efficacy and tumor weight in 32D-WT and 32D-T315I cells *in vivo* (**Figures 2B, C**). During the *in vivo* experiment, we did not observe any changes in body weight (**Figure 2D**). These results demonstrated that down-regulation of CBP potentially inhibited cell growth *in vitro* and *in vivo* in the BCR-ABL independent way.

### Knock-Down of CBP Activated p300 Downstream Pathway in CML Cells

**Figure 3:**
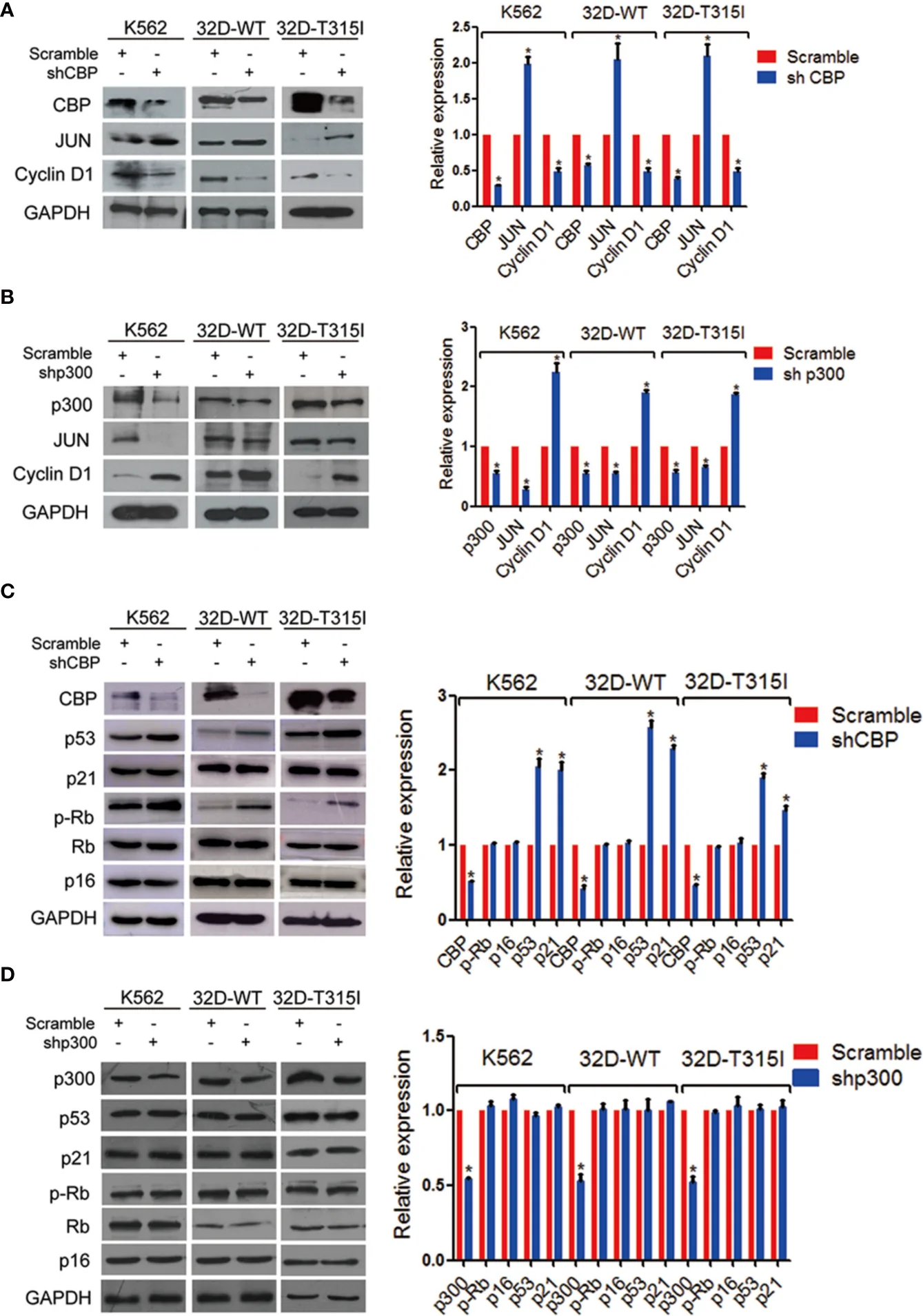
Down-regulation of CREB binding protein (CBP) increased senescent molecular expressions in chronic myeloid leukemia (CMLs) expressing wild-type or T315I mutant BCR-ABL. **(A)** The expression levels of JUN and cyclinD1 were evaluated by Western blot in K562, 32D-WT, and 32D-T315I cells following the down-regulation of CBP. **(B)** The expression levels of JUN and cyclinD1 were evaluated by Western blot in K562, 32D-WT, and 32D-T315I cells following the down-regulation of p300. **(C)** Cell senescence-related protein alterations were examined by Western blot in K562, 32D-WT, and 32D-T315I cells following the down-regulation of CBP. **(D)** Cell senescence-related protein alterations were examined by Western blot in K562, 32D-WT, and 32D-T315I cells following the down-regulation of p300. The data were analyzed using the Gel-Pro analyzer. The data represent mean ± SD derived from three independent experiments. Signiﬁcant changes are indicated as follows: *p < 0.05, compared to scramble group (unpaired student’s t-test (two groups).

As we described above, down-regulation of CBP promoted cell differentiation and senescence. To further investigate the mechanism of CBP inhibition on cell senescence, we detected the expression of CBP and p300 downstream molecules. We found that the knockdown of CBP decreased the expression of cyclinD1 which was one of the downstream molecules of β-catenin/CBP, but concomitantly increased the expression level of JUN which was a downstream molecule of β-catenin/p300, independent of the mutation status of BCR-ABL in CML cells (**Figure 3A**). In contrast, silence of p300 induced the up-regulation of cyclinD1 and down-regulation of JUN (**Figure 3B**). Therefore, CBP inhibition activated β-catenin/p300 signaling.

Since CBP inhibition inducedβ-catenin/p300 signaling mediated cell differentiation and finally led to cell senescence, subsequently, we evaluated if cell senescence was dependent on the p53/p21 and p16/p-Rb pathways, which play key roles in regulation of senescence in human fibroblasts and also participate directly in transcriptional regulation (20, 21). The expression of p53, p21, p-Rb, Rb, and p16 were detected by Western blot. The results indicated that silencing CBP resulted in a significant accumulation of p53 and p21 but did not alter the phosphorylated Rb and p16 expression level in both BCR-ABL wild-type and T315I mutant CMLs (**Figure 3C**). These indicate that the down-regulation of CBP induced cell senescence in a p53-dependent manner.

### Inhibition of CBP Increased the Binding of β-Catenin to p300 in CML Cells

**Figure 4:**
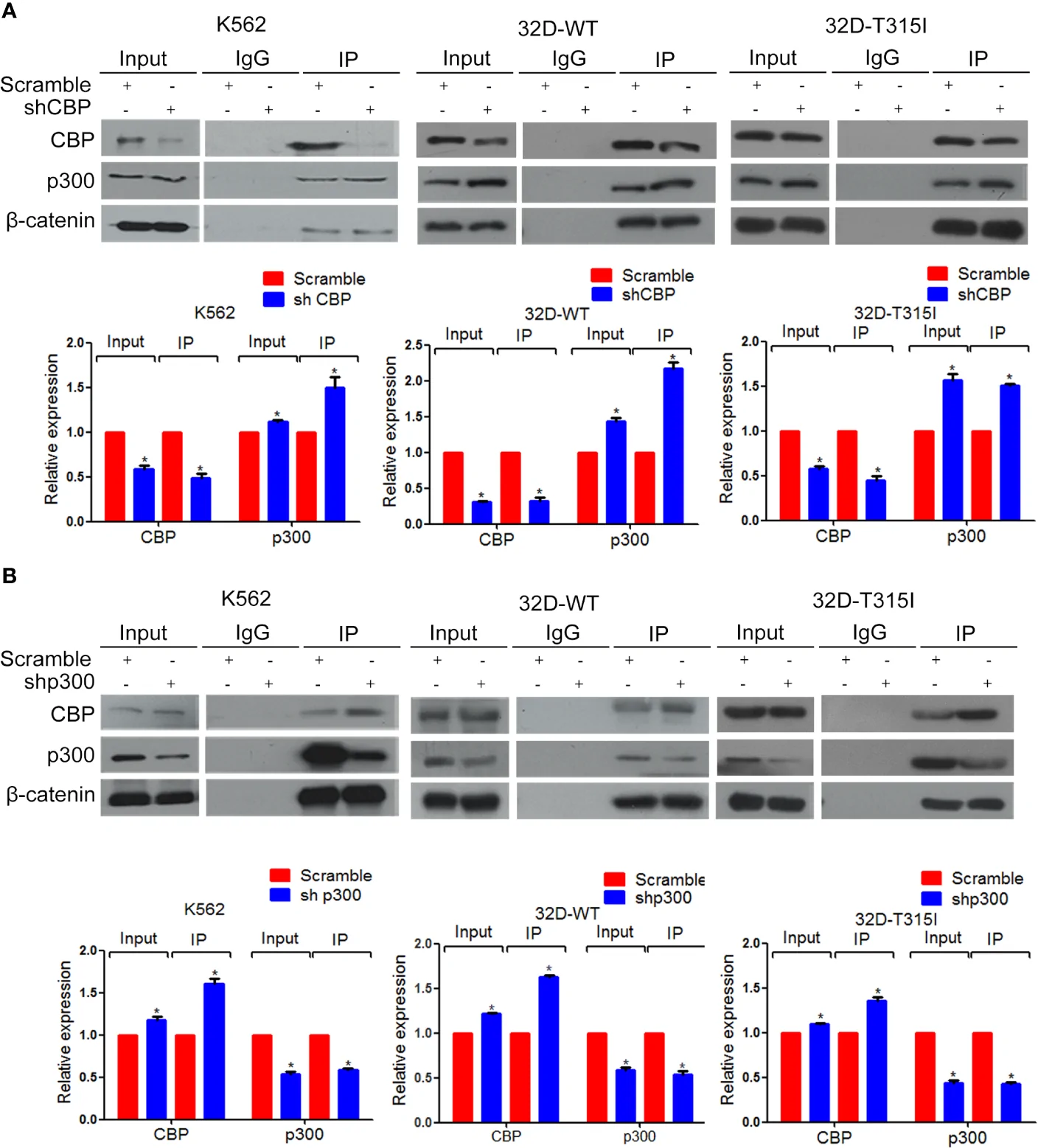
Enhancement of β-catenin binding to p300 by down-regulation of CREB binding protein (CBP). Co-immunoprecipitation assay was performed in K562, 32D-WT, 32D-T315I cells. **(A)** CBP down-regulation significantly increased the binding of β-catenin to p300. **(B)** Knock down of p300 increased the binding of β-catenin to CBP in K562, 32D-WT, 32D-T315I cells. IgG was used as a control. The data were analyzed using the Gel-Pro analyzer. The data represent mean ± SD derived from three independent experiments. Signiﬁcant changes are indicated as follows: *p < 0.05, compared to scramble group (One-Way ANOVA).

To further confirm the mechanism of inducing differentiation by CBP down-regulation, co-immunoprecipitation (Co-IP) was performed and indicated that the down-regulated expression of CBP increased the binding of β-catenin to p300 in both BCR-ABL wild-type and T315I mutant CMLs (**Figure 4A**). And vice versa, the down-regulation of p300 increased the binding of β-catenin to CBP (**Figure 4B**). According to the above research, we come to conclude that decrease of CBP significantly promote the recruitment of p300 by β-catenin, which consequently activated β-catenin/p300 mediated senescence pathway.

## Discussion

In recent years, a variety of TKIs targeting at BCR-ABL with increasing specificity and efficacy have been developed, which have fundamentally promoted the treatment of CML. However, the mutations especially the T315I mutation in BCR-ABL kinase domain have led to the development of drug resistance and hindered the success of imatinib or second generation TKIs in the treatment of CML. Worse still, increasing mutations induced by TKIs have been identified, which limited its use in clinic. Clinically, the successful use of differentiation therapies, such as all-trans retinoic acid (ATRA), has been successful in 5%–10% AML patients but not in CML patients (22). Under these conditions, we sought to find a new strategy for CML’s treatment on basis of cell growth and differentiation in HSCs. Our study demonstrates the potential of a CBP inhibitor therapy enhances of the binding of β-catenin to p300 which mediated cell differentiation and senescence in BCR-ABL-wild-type and -T315I mutant CML. We found that the inhibition of CBP is efficacious in activating β-catenin/p300 signaling, which promoted cell differentiation and induces p53/p21 dependent senescence *in vitro* and *in vivo*.

Wnt signaling pathways regulate a variety of processes including cell growth, oncogenesis, and development. Upon Wnt signaling, β-catenin accumulates in the nucleus and binds to transcription factors of the TCF/LEF family to activate transcription and constitutive activation of downstream target genes, such as surviving, c-myc and cyclinD1 by the TCF/LEF-β-catenin complex, which is essential for balancing cell survival and differentiation (8–10, 14, 23, 24). CBP and p300, as transcriptional coactivators, bind to the COOH-terminal region of β-catenin. β-catenin physically interact with the CREB-binding domain of CBP/p300 to activate transcription in mammalian cells (25). CBP and p300, recruited by β-catenin, have individually been identified as co-activators in self-renewal and differentiation in HSCs (8–10, 23). These implicate the enhancement of β-catenin recruiting p300 maybe a novel treatment strategy to eradicate CML expressing either wild type or mutated BCR-ABL.

Our results indicate that β-catenin recruits more p300 after CBP expression is silenced in CMLs expressing wild-type and T315I mutant BCR-ABL. The repression of CBP decreased the expression of cyclinD1 (26), which is highly coordinated with growth inhibition and cell differentiation (27–29). Moreover, the suppression of CBP switches the balance to β-catenin/p300 mediated cell differentiation, suggesting an entirely novel approach for the treatment of CML, with the therapeutic potential to treat BCR-ABL-T315I mutant CML. However, the mechanism by which β-catenin/p300 signaling mediate cell differentiation remains to be elucidated.

Cellular senescence disables proliferation in damaged cells and is relevant for cancer and aging. This involvement of tumor-suppressor pathways led to the identification of senescence as potent tumor-suppressor mechanism. Several studies have revealed that, adult stem cells could escape cellular senescence by avoiding both differentiation and the activation of checkpoint responses that arrest the cell cycle in cell homeostasis. The key proteins that induce senescence are members of the p53/p21 and p16/p-Rb tumor suppressor networks that function to arrest proliferation and contribute to the irreversibility of the senescent state. In our study, we used SA-β-Gal staining to identify senescent cells because it is a proven and reliable marker of senescence in many settings. To determine whether p53/p21 or p16/p-Rb were required for β-catenin/p300 mediated senescence, we examined these markers and mediators of senescence. The results indicated that the increased β-catenin/p300 mainly activated p53/p21 signaling mediated cell senescence. This offers a possibility to promote cell differentiation and senescence and limit proliferation *via* a switch to β-catenin binding to p300 by targeting CBP, and to ultimately eliminate CMLs, including expressing T315I mutant BCR-ABL CMLs.

**Figure 5:**
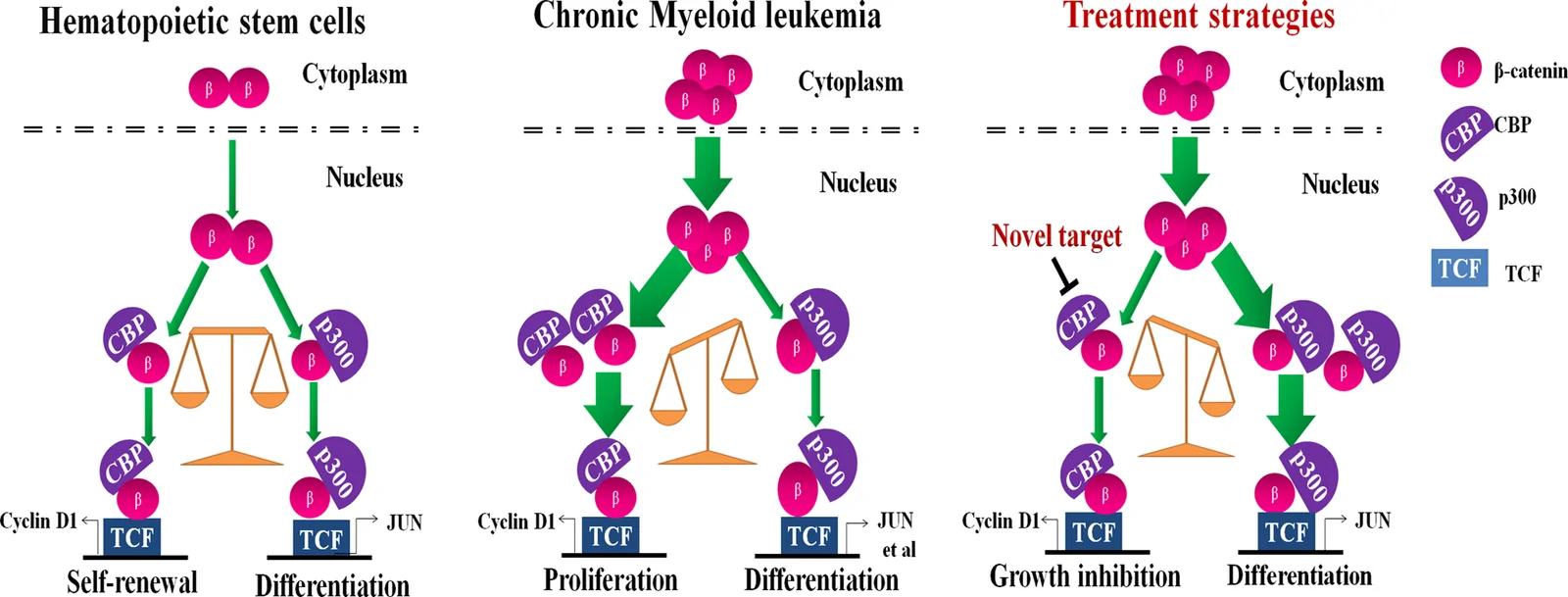
A schematic model illustrating the putative mechanism by which CREB binding protein (CBP) down-regulation inhibits cell proliferation and induces cell differentiation. The interaction of β-catenin with CBP or p300crucially switches to hematopoietic stem cell (HSC) cells going to proliferation or differentiation, respectively. HSCs continuously provide more differentiated cells, while maintaining the HSC pool size throughout life by balancing self-renewal and differentiation (Left). However, β-catenin and CBP are aberrantly expressed in chronic myeloid leukemia (CML), switching the balance to malignant proliferation (Middle). The inhibition of CBP effectively inhibited cell growth and shifted the balance to β-catenin/p300-mediated senescence signaling in CML cells with expressing the wild-type or T315I mutant BCR-ABL (Right).

These results suggest the binding of β-catenin to CBP or p300 decides CML cell fate, proliferation or differentiation, which is independent of BCR-ABL mutation status. The inhibition of CBP could be a promising strategy for the treatment of CMLs including BCR-ABLT315I mutant CMLs (**Figure 5**). We speculate that effect may be related with a response to the balance mechanism of β-catenin/CBP and β-catenin/p300. However, the specific mechanism is currently unknown and deserve further investigation.

## Data Availability Statement

The raw data supporting the conclusions of this article will be made available by the authors, without undue reservation.

## Ethics Statement

The animal study was reviewed and approved by Institutional Animal Care and Use Committee of Sun Yat-Sen University.

## Author Contributions

LF designed the research. KY, FW, and HZ performed the *in vitro* experiments and analyzed data. XKW, LC, XS, XPW, and QH performed the animal experiments. ZC analyzed data and provided technical assistance. KY wrote the paper. LF and Z-SC provided technical assistance and revised the manuscript. All authors contributed to the article and approved the submitted version.

## Funding

This work was financially supported by the National Science & Technology Major Project Key New Drug Creation and Manufacturing Program, China (2018ZX09711002), the Natural Scientific Foundation of China (No. 82073882, No. 81673463), the Guangdong Provincial Special Fund for Marine Economic Development Project (GDNRC [2020]042), the Science and Technology Foundation of Guangdong Province (No, 2016A030312014), and the Scientific and Technological Leading Talent Project of Guangdong Province (2015, China). 

## Conflict of Interest

Author QH was employed by Guangzhou Handy Biotechnological Co., Ltd.

The remaining authors declare that the research was conducted in the absence of any commercial or financial relationships that could be construed as a potential conflict of interest.

## Correction note

A correction has been made to this article. Details can be found at: 10.3389/fonc.2026.2001990.

## References

[1] Chen Y Peng C Li D Li S. Molecular and cellular bases of chronic myeloid leukemia. Protein Cell (2010) 1(2):124–32. doi: 10.1007/s13238-010-0016-z, PMID: 21203982 PMC4875160

[2] Weisberg E Manley PW Breitenstein W Bruggen J Cowan-Jacob SW Ray A. Characterization of AMN107, a selective inhibitor of native and mutant Bcr-Abl. Cancer Cell (2005) 7(2):129–41. doi: 10.1016/j.ccr.2005.01.007, PMID: 15710326

[3] Talpaz M Shah NP Kantarjian H Donato N Nicoll J Paquette R. Dasatinib in imatinib-resistant Philadelphia chromosome-positive leukemias. New Engl J Med (2006) 354(24):2531–41. doi: 10.1056/NEJMoa055229, PMID: 16775234

[4] Gorre ME Mohammed M Ellwood K Hsu N Paquette R Rao PN. Clinical resistance to STI-571 cancer therapy caused by BCR-ABL gene mutation or amplification. Science (2001) 293(5531):876–80. doi: 10.1126/science.1062538, PMID: 11423618

[5] Blay JY von Mehren M. Nilotinib: a novel, selective tyrosine kinase inhibitor. Semin Oncol (2011) 38(Suppl 1):S3–9. doi: 10.1053/j.seminoncol.2011.01.016, PMID: 21419934 PMC4004101

[6] Hanfstein B Muller MC Kreil S Ernst T Schenk T Lorentz C. Dynamics of mutant BCR-ABL-positive clones after cessation of tyrosine kinase inhibitor therapy. Haematologica (2011) 96(3):360–6. doi: 10.3324/haematol.2010.030999, PMID: 21134983 PMC3046266

[7] Khorashad JS Kelley TW Szankasi P Mason CC Soverini S Adrian LT. BCR-ABL1 compound mutations in tyrosine kinase inhibitor-resistant CML: frequency and clonal relationships. Blood (2013) 121(3):489–98. doi: 10.1182/blood-2012-05-431379, PMID: 23223358 PMC3548169

[8] Takemaru KI Moon RT. The transcriptional coactivator CBP interacts with beta-catenin to activate gene expression. J Cell Biol (2000) 149(2):249–54. doi: 10.1083/jcb.149.2.249, PMID: 10769018 PMC2175158

[9] Teo JL Ma H Nguyen C Lam C Kahn M. Specific inhibition of CBP/beta-catenin interaction rescues defects in neuronal differentiation caused by a presenilin-1 mutation. Proc Natl Acad Sci USA (2005) 102(34):12171–6. doi: 10.1073/pnas.0504600102, PMID: 16093313 PMC1189325

[10] Rebel VI Kung AL Tanner EA Yang H Bronson RT Livingston DM. Distinct roles for CREB-binding protein and p300 in hematopoietic stem cell self-renewal. Proc Natl Acad Sci USA (2002) 99(23):14789–94. doi: 10.1073/pnas.232568499, PMID: 12397173 PMC137497

[11] Zhang Y Wang S Kang W Liu C Dong Y Ren F. CREPT facilitates colorectal cancer growth through inducing Wnt/beta-catenin pathway by enhancing p300-mediated beta-catenin acetylation. Oncogene (2018) 37(26):3485–500. doi: 10.1038/s41388-018-0161-z, PMID: 29563608 PMC6021369

[12] Giebel B Bruns I. Self-renewal versus differentiation in hematopoietic stem and progenitor cells: a focus on asymmetric cell divisions. Curr Stem Cell Res Ther (2008) 3(1):9–16. doi: 10.2174/157488808783489444, PMID: 18220918

[13] Seita J Weissman IL. Hematopoietic stem cell: self-renewal versus differentiation. Wiley Interdiscip Rev Syst Biol Med (2010) 2(6):640–53. doi: 10.1002/wsbm.86, PMID: 20890962 PMC2950323

[14] Ma H Nguyen C Lee KS Kahn M. Differential roles for the coactivators CBP and p300 on TCF/beta-catenin-mediated survivin gene expression. Oncogene (2005) 24(22):3619–31. doi: 10.1038/sj.onc.1208433, PMID: 15782138

[15] Zhao C Blum J Chen A Kwon HY Jung SH Cook JM. Loss of beta-catenin impairs the renewal of normal and CML stem cells in vivo. Cancer Cell (2007) 12(6):528–41. doi: 10.1016/j.ccr.2007.11.003, PMID: 18068630 PMC2262869

[16] Wang Y Krivtsov AV Sinha AU North TE Goessling W Feng Z. The Wnt/beta-catenin pathway is required for the development of leukemia stem cells in AML. Science (2010) 327(5973):1650–3. doi: 10.1126/science.1186624, PMID: 20339075 PMC3084586

[17] Heidel FH Bullinger L Feng Z Wang Z Neff TA Stein L. Genetic and pharmacologic inhibition of beta-catenin targets imatinib-resistant leukemia stem cells in CML. Cell Stem Cell (2012) 10(4):412–24. doi: 10.1016/j.stem.2012.02.017, PMID: 22482506 PMC3339412

[18] Coluccia AM Vacca A Dunach M Mologni L Redaelli S Bustos VH. Bcr-Abl stabilizes beta-catenin in chronic myeloid leukemia through its tyrosine phosphorylation. EMBO J (2007) 26(5):1456–66. doi: 10.1038/sj.emboj.7601485, PMID: 17318191 PMC1817619

[19] Collins SJ Ruscetti FW Gallagher RE Gallo RC. Normal functional characteristics of cultured human promyelocytic leukemia cells (HL-60) after induction of differentiation by dimethylsulfoxide. J Exp Med (1979) 149(4):969–74. doi: 10.1084/jem.149.4.969, PMID: 219131 PMC2184853

[20] Collado M Serrano M. The power and the promise of oncogene-induced senescence markers. Nat Rev Cancer (2006) 6(6):472–6. doi: 10.1038/nrc1884, PMID: 16723993

[21] Campisi J d’Adda di Fagagna F. Cellular senescence: when bad things happen to good cells. Nat Rev Mol Cell Biol (2007) 8(9):729–40. doi: 10.1038/nrm2233, PMID: 17667954

[22] Wald DN Vermaat HM Zang S Lavik A Kang Z Peleg G. Identification of 6-benzylthioinosine as a myeloid leukemia differentiation-inducing compound. Cancer Res (2008) 68(11):4369–76. doi: 10.1158/0008-5472.CAN-07-6559, PMID: 18519698 PMC3896053

[23] Li J Sutter C Parker DS Blauwkamp T Fang M Cadigan KM. CBP/p300 are bimodal regulators of Wnt signaling. EMBO J (2007) 26(9):2284–94. doi: 10.1038/sj.emboj.7601667, PMID: 17410209 PMC1864967

[24] Gang EJ Hsieh YT Pham J Zhao Y Nguyen C Huantes S. Small-molecule inhibition of CBP/catenin interactions eliminates drug-resistant clones in acute lymphoblastic leukemia. Oncogene (2014) 33(17):2169–78. doi: 10.1038/onc.2013.169, PMID: 23728349 PMC3994178

[25] Goodman RH Smolik S. CBP/p300 in cell growth, transformation, and development. Genes Dev (2000) 14(13):1553–77. doi: 10.1101/gad.14.13.1553, PMID: 10887150

[26] Tetsu O McCormick F. Beta-catenin regulates expression of cyclin D1 in colon carcinoma cells. Nature (1999) 398(6726):422–6. doi: 10.1038/18884, PMID: 10201372

[27] Canzoniere D Farioli-Vecchioli S Conti F Ciotti MT Tata AM Augusti-Tocco G. Dual control of neurogenesis by PC3 through cell cycle inhibition and induction of Math1. J Neurosci: Off J Soc Neurosci (2004) 24(13):3355–69. doi: 10.1523/JNEUROSCI.3860-03.2004, PMID: 15056715 PMC6730030

[28] Mori J Takahashi-Yanaga F Miwa Y Watanabe Y Hirata M Morimoto S. Differentiation-inducing factor-1 induces cyclin D1 degradation through the phosphorylation of Thr286 in squamous cell carcinoma. Exp Cell Res (2005) 310(2):426–33. doi: 10.1016/j.yexcr.2005.07.024, PMID: 16153639

[29] Kim ES Lee JJ Wistuba II. Cotargeting cyclin D1 starts a new chapter in lung cancer prevention and therapy. Cancer Prev Res (2011) 4(6):779–82. doi: 10.1158/1940-6207.CAPR-11-0143, PMID: 21636543

