# Correction: Target inhibition of CBP induced cell senescence in BCR-ABL- T315I mutant chronic myeloid leukemia

**DOI:** 10.3389/fonc.2026.2001990

**Published:** 2026-10-22

**Authors:** Ke Yang, Fang Wang, Hong Zhang, Xiaokun Wang, Likun Chen, Xiaodong Su, Xingping Wu, Qianqian Han, Zhen Chen, Zhe-Sheng Chen, Liwu Fu

**Affiliations:** 1 Sun Yat-sen University Cancer Center, State Key Laboratory of Oncology in Southern China, Collaborative Innovation Center for Cancer Medicine, Guangzhou, China; 2 Department of Research and Development, Guangzhou Handy Biotechnological Co., Ltd., Guangzhou, China; 3 Department of Pharmaceutical Sciences, College of Pharmacy and Health Sciences, St. John’s University, Queens, NY, United States

**Keywords:** CREB binding protein, cell differentiation, cell senescence, chronic myeloid leukemia, tyrosine kinase inhibitors resistance

There was a mistake in **Figure 1E** as published. During figure preparation, a file-selection error resulted in the 32D-WT micrograph being mistakenly used for the 32D-T315I panels in **Figure 1E**.

**Figure 1:**
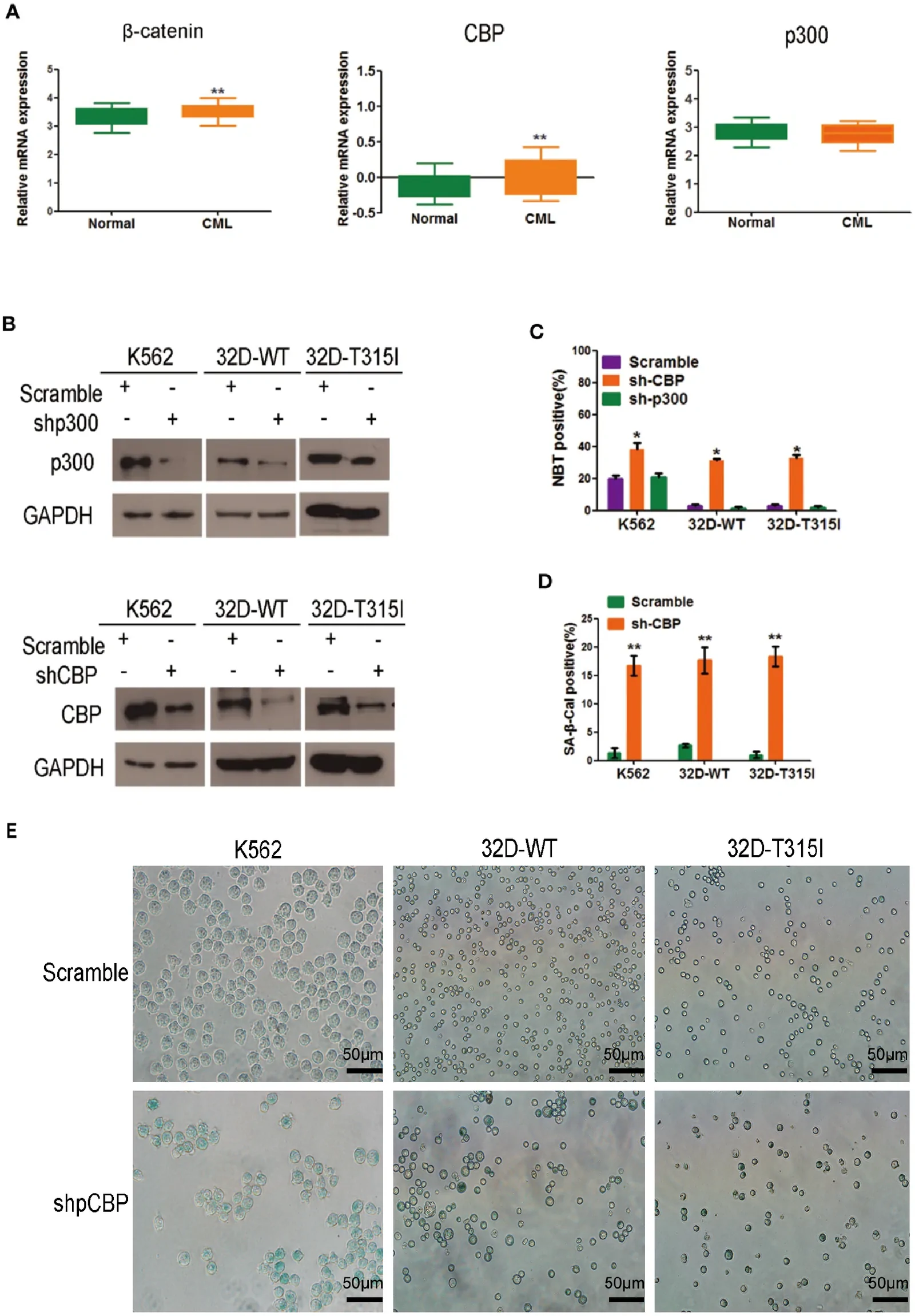
Effect of CREB binding protein (CBP) down-regulation on cell differentiation and scenescence in chronic myeloid leukemia (CMLs) expressing wild-type or T315I mutant BCR-ABL. **(A)** In silico analysis of β-catenin, CBP, and p300 expression in human CML. Data was analyzed using publicly available Oncomine database (www.oncomine.org); *p < 0.05; **p < 0.01 (Student’s t-test). **(B)** Efficient knocks down CBP or p300 in K562, 32D-WT, 32D-T315I cells as revealed by Western blot. **(C)** Differentiation status was determined by nitrobluetetrazolium (NBT) reduction assay in K562, 32D-WT, and 32D-T315I cells following the down-regulation of CBP or p300. **(D)** Cell senescence status was determined in cells with the indicated vector (scramble and sh-CBP) transfection for 72 h by the SA-β-Gal staining assay. The values are evaluated using the SA-β-Gal positive ratio. **(E)** Senescent cells were identified as blue-stained cells under an Olympus IX71 inverted microscope. Representative images of senescent cells were taken by Olympus IX71 inverted microscope. The data represent Mean ± SEM derived from three independent experiments. Significant changes are indicated as follows: *p < 0.05; **p < 0.01, compared to scramble group (unpaired student’s t-test).

This correction does not alter the quantitative data presented in **Figure 1D**, statistical analyses, experimental results, or the core conclusions of the original article. The corrected **Figure 1** appears below.

The original version of this article has been updated.

